# Distribution and population structure of the smooth‐hound shark, *Mustelus mustelus* (Linnaeus, 1758), across an oceanic archipelago: Combining several data sources to promote conservation

**DOI:** 10.1002/ece3.9098

**Published:** 2022-07-13

**Authors:** Fernando Espino, José Antonio González, Néstor E. Bosch, Francisco J. Otero‐Ferrer, Ricardo Haroun, Fernando Tuya

**Affiliations:** ^1^ Research Group in Biodiversity and Conservation, IU‐ECOAQUA, Scientific and Technological Marine Park Universidad de Las Palmas de Gran Canaria Telde Canary Islands Spain; ^2^ Grupo de Investigación en Ecología Marina Aplicada y Pesquerías, Facultad de Ciencias del Mar Universidad de Las Palmas de Gran Canaria Las Palmas de Gran Canaria Canary Islands Spain; ^3^ The UWA Oceans Institute, School of Biological Sciences The University of Western Australia Crawley Western Australia Australia

**Keywords:** Atlantic Ocean, Canary Islands, Chondrichthyes, elasmobranchs, endangered species, macroecology

## Abstract

Sharks play a key role in the structure and functioning of marine ecosystems. More ecological information is essential to implement responsible management and conservation actions on this fauna, particularly at a regional level for threatened species. *Mustelus mustelus* is widely found in the eastern Atlantic Ocean and catalogued as “Vulnerable” by the IUCN European assessment. In this study, data on the distribution and population structure of this species across the islands of the Canarian archipelago, located along an east to west gradient in the north‐eastern Atlantic, were collected by taking advantage of “Local Ecological Knowledge,” in terms of sightings in coastal waters and long‐term imprints on the local gastronomic heritage, and decadal fisheries landings. Both sources of quantitative data (sightings and fisheries landings) demonstrated that adults of *M. mustelus* has a significantly larger presence in the eastern and central, than in the western islands of the archipelago. This is also reflected on local gastronomic legacies, with a larger number of recipes in the eastern and central islands. Adult smooth‐hound sharks were significantly more observed in sandy and sandy‐rocky bottoms, with individuals seen throughout the entire year, whereas juveniles aggregate on very shallow waters in spring and summer. Such aggregations require a special management strategy, as they play a key role in critical life stages; these sites should be protected from human perturbations. We also suggest a temporal fishing ban between April and October, when individuals tend to concentrate on nearshore waters. Because of the large differences in presence of this shark among the Canary Islands, management of the species should be adapted to the specific peculiarities of each island, rather than adopting a management policy at the entire archipelago‐scale. Overall, this study sets the basis for further investigation to promote conservation of this vulnerable shark in the study region.

## Graphical abstract

This work combines three sources of data to investigate the distribution and population structure of a threatened shark species.

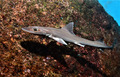

## 1 INTRODUCTION

Sharks are a diverse group of fishes, including 536 species within the class Elasmobranchii (Ebert et al., 2021), which are found in oceanic and coastal waters, and even in freshwater‐influenced systems (Dulvy et al., 2017). Generally, the term shark is associated with large‐sized species, typically apex predators at the top of food webs; however, most sharks are mesotrophic predators less than 2 m in size, which mostly live on the continental shelf (Bizzarro et al., 2017). Sharks play a key role in the structure and functioning of marine ecosystems via predation (Heithaus et al., 2008), connecting food webs across habitats, and spreading predation risk vertically and horizontally across seascapes (Dulvy et al., 2017; Lester et al., 2020). Despite their ecological importance, sharks are one of the most endangered groups of marine species worldwide (Bräutigam et al., 2015; Cortés, 2002; Dulvy et al., 2014, 2021; Pacoureau et al., 2021), with many populations experiencing severe declines due to anthropogenic pressures. The major threat is overexploitation through fisheries and incidental catches (bycatch), followed by habitat destruction, pollution, and climate change (Bräutigam et al., 2015; Dulvy et al., 2014; Seitz & Poulakis, 2006; Worm et al., 2013). Certain life history traits, including late sexual maturity, gestation period, low fecundity, slow growth and longevity, exacerbates their vulnerability to the above‐mentioned impacts (Rodrigues et al., 2019). From a total of 465 sharks species recently assessed by the International Union for Conservation of Nature (IUCN) (https://www.iucnredlist.org/ [accessed February 15, 2022]), 74 (15.9%) are included in the following threatened categories established in the Red List: 11 (2.36%) Critically Endangered, 15 (3.22%) Endangered, and 48 (10.32%) Vulnerable. Without a doubt, more ecological information on these taxa is essential to guide management and conservation actions (Dulvy et al., 2014), particularly for the north‐eastern Atlantic, where sharks face alarming levels of extinction risk (Walls & Dulvy, 2020).

When ecological data are sparse, difficult, and expensive to obtain, “Local Ecological Knowledge” (LEK) provide an alternative, which has proved successful for a range of endangered marine species, such as cetaceans (Turvey et al., 2013), seahorses (Heard et al., 2019; Otero‐Ferrer et al., 2017), and sharks (Hiddink et al., 2019; Leduc et al., 2021). LEK approaches are often based on surveys that target population sectors directly interacting with species; for example, fishers or divers in the case of coastal species (Leduc et al., 2021). The imprint of social knowledge, however, may arise from activities that do not directly interact with species and transcend multiple human generations. For example, regional gastronomy is determined by the availability of raw materials and results from interactions between natural (geological, geographical, biological, etc.) conditions and historical events (Almenar, 2020; Coll et al., 2010). This idea applies to both domestic and fine cuisine, including that underpinned by products derived from local and regional coastal fisheries through the last centuries such as black scabbardfish, *Aphanopus carbo* Lowe, 1839 (Maul, 1950) and cod, *Gadus morhua* Linnaeus, 1758 (Kurlansky, 2013). Seafood products caught by artisanal fisheries in nearshore waters are often carried out by small‐sized boats of limited power; this is the case of oceanic archipelagos, such as the Canary Islands (González, 1991; González, González‐Lorenzo, et al., 2020). As a result, a seafood gastronomy based on local products may somehow reflect the availability of species through multiple generations (da Silva et al., 2015).

**FIGURE 1:**
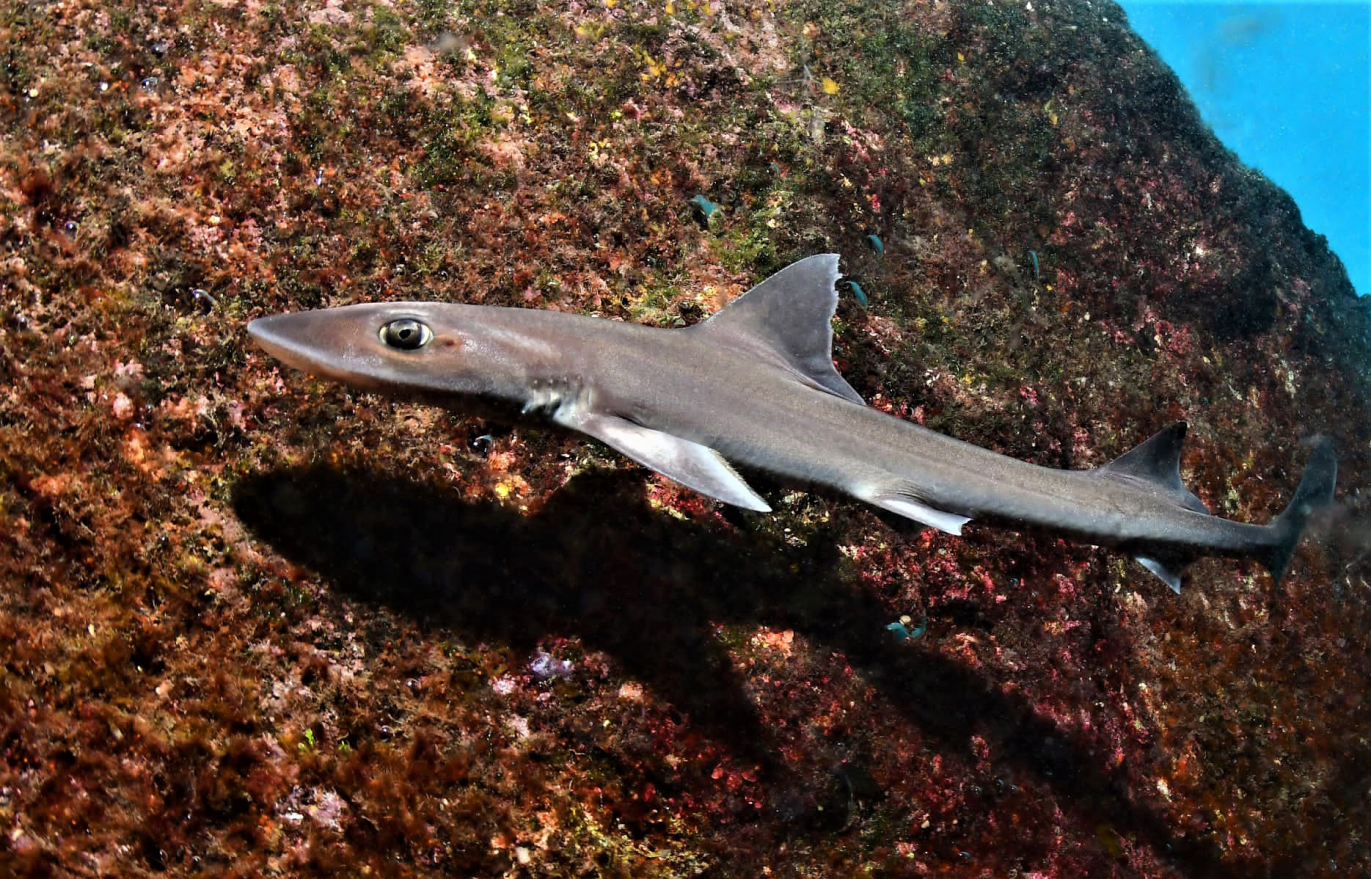
Juvenile of *Mustelus mustelus* in a shallow rocky bottom in the island of Gran Canaria, Canary Islands. Photograph taken by Alfredo Ubierna. Reproduced with permission of the author

The smooth‐hound, *Mustelus mustelus* (Linnaeus, 1758) (Carcharhiniformes: Triakidae) (Figure 1), is a small‐sized shark, typically between 100 and 150 cm in total length, that may reach up to 200 cm of total length (Reiner, 1996; Sanches, 1991). It is a demersal fish that usually swims near the sea bottom, from the intertidal down to 350 m depth, with most observations and captures reported between 5 and 100 m depth, generally on sandy bottoms, but occasionally on muddy or detritic bottoms (Capapé et al., 2006). This shark is a predatory species feeding on crustaceans, cephalopods, and small bony fishes (Compagno, 1984). The species has a widespread distribution in the eastern Atlantic. The presence of *M. mustelus* in the North Sea and the British Isles is not clear (ICES, 2021); no confirmed specimens have been found in northern parts of the ICES area in recent years, and historical records are questionable, especially those north of the Bay of Biscay. Information and data from northern Europe referring to *M. mustelus* likely refer to *Mustelus asterias* Cloquet, 1821, and separating these two species is unreliable in the North Sea (Compagno et al., 2005; Farrell et al., 2009; ICES, 2021). *M. mustelus* is distributed across western Africa down to South Africa, and towards the southwestern coasts of the Indian Ocean; it is also found in the Mediterranean Sea and several oceanic eastern‐Atlantic archipelagos: Madeira, Canary Islands, Cape Verde, and São Tomé and Príncipe (Compagno et al., 2005). In the Mediterranean, southern Europe and western African coasts, the species is targeted by bottom trawling, hook‐and‐line gears and bottom trammel nets. The species is consumed fresh, frozen, dried, and salted and smoked, and the liver is used for oil production (Compagno, 1984). This shark has been catalogued as ‘Vulnerable’ by the IUCN European regional assessment (Farrell et al., 2015). However, some recent studies have shown abrupt declines in the abundance of certain populations, calling for an urgent revision on its conservation category; for example, in the Mediterranean Sea, smooth‐hound sharks have declined by 80–90% since the beginning of last century, disappearing in a large part of their original distributional range during the 1980s and 1990s (Colloca et al., 2017). Research efforts to assess the status of sharks and, in particular to identify those that are threatened, are essential in any conservation planning (Meyers et al., 2017; Simpfendorfer et al., 2011).

In the Canary Islands, three species within the Triakidae are found (*Galeorhinus galeus* (Linnaeus, 1758), *Mustelus mustelus*, and *M. asterias*), and *M. mustelus* is, by far, the most abundant species of the family, particularly in coastal waters (Brito et al., 2002; González, González‐Lorenzo, et al., 2020), which is popularly known as “cazón”. This shark lives mainly on soft bottoms (Brito et al., 2002), and there is no information about the temporal trend of the stock. In the archipelago, there has been a long tradition in the consumption of triakid species. Between the XVth and XVIIth centuries, after the annexation of the Canary Islands to the Kingdom of Castile, settlers coming from the Iberian Peninsula, mostly from Andalusia, carried out a large fishing activity in the nearby fishing grounds at the north‐western African coasts (Rumeu de Armas, 1977). In those times, fishery resources were largely preserved on‐board (dried and salted) (Balguerías, 1993), including sharks of the Triakidae family, which were targeted and well appreciated in the Andalusian cuisine, including several regional recipes for this fish, and so reflecting the cultural gastronomic heritage of the archipelago. Currently, *M. mustelus* is not included in the National Catalogue of Protected Species of Spain (Law 139/2011). Similarly, the species is not protected at the regional level (Autonomous Government of the Canary Islands, Law 4/2010). At the fisheries level, captures of this shark are permitted. At present, there is no study on whether fisheries on this shark are sustainable through time. In general, however, coastal fishery resources of the Canary Islands are severely declining due to overexploitation (Castro et al., 2019).

In this study, we aimed to assess if patterns in the spatial distribution of the smooth‐hound, *M. mustelus*, across the Canary Islands (eastern Atlantic) is reflected by LEK, in terms of sightings in coastal waters and long‐term imprints on the local gastronomic heritage, as well as by fisheries landings. This approach allowed us to gather data on the population structure (depth, sizes, seasons, and habitats) where this shark occurs, including observation of aggregation events. In brief, this study sets the basis for further investigations to promote conservation of this shark species in the study region.

## 2 MATERIAL AND METHODS

### 2.1 Study area

The Canarian archipelago, in the eastern Atlantic off the Northwest African coast, comprises seven main islands and several islets that have emerged after successive volcanic events. Altogether, the islands have a surface area of ca. 7435 km^2^ and a coastline covering ca. 1290 km (Fernández‐Palacios & Whitaker, 2008). The easternmost island (Fuerteventura) lies at only ca. 95 km away from western African mainland, whereas the island of La Palma is almost at ca. 416 km from the African coast (Fernández‐Palacios & Martín Esquivel, 2001). Differences in the composition and abundance of marine biodiversity across the entire archipelago have been previously reported for macroalgae (Tuya & Haroun, 2009), reef fishes (Tuya et al., 2004) and rays (Tuya et al., 2021). In general, species of temperate affinities are limited (e.g., *Sparus aurata*) or more abundant (e.g., *Coris julis* and *Serranus papilionaceus*) in the easternmost islands, while species of tropical affinities are limited (e.g., *Corniger spinosus* and *Gymnothorax miliaris*) or more abundant (e.g., *Aulostomus strigosus* and *Heteropriacanthus fulgens*) in the westernmost islands (Brito et al., 2001). It has long been considered that this is a result of large‐scale oceanographic variation associated with the proximity of the Canary Islands to the continental shores of Africa, with the eastern islands regularly influenced by the seasonal upwelling off the African coast (Davenport et al., 2002). In turn, the westernmost islands (La Palma and El Hierro) often have a higher sea surface temperature (ca. 2°C) and lower productivity (ca. 237 vs. 145 g C m^−2^ yr^−1^) than the easternmost islands (Lanzarote and Fuerteventura) (Barton et al., 1998; Davenport et al., 2002).

All islands are volcanic, with different ages and geological histories, which have translated into differences in their geomorphology (Fernández‐Palacios & Martín Esquivel, 2001) (Table 1). Each island has arisen from an independent volcanic system, except the easternmost islands (Fuerteventura and Lanzarote), which share the same insular shelf, and are separated by a narrow strait with a maximum depth of ca. 50 m. The rest of the islands are separated by deep waters with depths ranging between 2000 and 3000 m (Acosta et al., 2003). In this study, in terms of data analysis, islands were sorted into three groups, following an east‐to‐west gradient of varying proximity to the African coast. This arrangement corresponds to similarities in the geological histories and relevant geomorphological features of islands, following a mantle‐plume “hotspot” volcanic origin (Table 1), while accounting for the oceanographic gradient across the archipelago. Lanzarote and Fuerteventura, including islets north of Lanzarote, were categorized as the “eastern islands.” The “central islands” include Gran Canaria and Tenerife, old to middle‐age islands with moderately large, and independent, insular shelfs. Finally, the islands of La Gomera, La Palma and El Hierro, that is, the “western islands,” are the youngest islands, particularly El Hierro and La Palma, which are characterized by small and abrupted insular shelfs (Table 1, Tuya et al., 2021). Three marine protected areas are found across the archipelago, including “Punta La Restinga‐Mar de Las Calmas” (El Hierro Island, from 1996), “Isla de La Graciosa e islotes del norte de Lanzarote” (northern Lanzarote Island, from 1998) and “La Palma” (La Palma Island, from 2001) (Tuya, García‐Díez, et al., 2006). The core areas of these reserves, where all fishing is banned, however, do not include adequate habitats for *M. mustelus*, and no special regulations in terms of fishing for this shark exist within these reserves. There are also 24 marine “Special Areas of Conservation” within the EU Natura 2000 network. However, there are no specific conservation measures for *M. mustelus* within these areas (Spanish Government Order ARM/2417/2011) and, consequently, fishing activities that could catch *M. mustelus* continue in these protected areas. The number of artisanal boats has remained stable through the last two decades in the Canary Islands, after a considerable reduction between the 1950s and the 1990s (Table 1, Castro et al., 2019). Similarly, the overall catch per unit effort (CPUE) of the demersal artisanal fishery in the Canary Islands has remained stable in the time period our data were collected.

**TABLE 1:** Geological and geomorphological characteristics of the Canary Islands (^1^islands, ^2^islets): Age (millions of years), island area (km^2^), island perimeter (km), and distance to the continent (km), based on information provided by Fernández‐Palacios and Martín Esquivel (2001) and our own data collection (https://visor.grafcan.es/visorweb/). The mean insular shelf width (km), the insular shelf area (km^2^) and the proportion (%) of hard and soft bottoms were calculated between the 0 m (sea level) and the 50 m depth isobath. The number of professional fishing ships, and human population (number of islanders per coastal perimeter, *n*° km^−1^; number of tourists per coastal perimeter and year, *n*° km^−1^ y^−1^ in brackets) are included

Island	Age	Island area	Island perimeter	Distance to the continent	Mean shelf width	Insular shelf area	Hard bottoms	Soft bottoms	Number of fishing ships^b^	Human population^c^
El Hierro^1^	0.8	267.81	95	383	0.23	31.91	72	28	15	119 (−)
La Palma^1^	1.5	706.85	126	416	0.46	87.27	37	63	36	662 (2976)
La Gomera^1^	12	367.87	87	333	0.71	83.96	24	76	23	250 (−)
Tenerife^1^	7.5	2032.93	269	284	0.67	280.12	41	59	265	3450 (22,115)
Gran Canaria^1^	14.5	1558.26	197	196	1.42	412.13	16	84	375	4328 (22,892)
Fuerteventura^1^	20.5	1651.92	255	95	1.65^a^	1119.80^a^	45	55	51	469 (8836)
Lobos^2^	0.05	4.53	9	123			‐	‐	‐	‐
Lanzarote^1^	15.5	805.88	203	125			43	57	52	769 (15,095)
La Graciosa^1^	0.04	27.31	28	151			‐	‐	8	‐
Roque del Oeste^2^	‐	0.02	0.73	‐			‐	‐	‐	‐
Montaña Clara^2^	0.03	1.38	4	159			‐	‐	‐	‐
Alegranza^2^	0.04	10.52	14	168	‐	13.48	‐	‐	‐	‐
Roque del Este^2^	‐	0.06	1.57	‐	‐	2.34	‐	‐	‐	‐

^a^ Lanzarote, Fuerteventura, Lobos, La Graciosa, Montaña Clara and Roque del Oeste share the same insular shelf.

^b^ Number of fishing ships according official data of the Canarian Government (see Tuya, Sánchez‐Jerez, & Haroun, 2006).

^c^ Human population: Number of islanders correspond to year 2021, and number of tourists correspond to year 2018 (before the SARS‐CoV‐2 pandemia), both according with official data of the Instituto Canario de Estadística (ISTAC, www.gobiernodecanarias.org/istac).

### 2.2 Shark presence and population structure through LEK: Sightings

We interviewed (*N* = 142) recreational angling fishers, spearfishers, commercial and recreational divers, marine scientists, underwater photographers, and managers of diving centers, who provided information about their shark observations between 1980 and 2020 ([Link], [Link]). All survey respondents were experienced (>20 years of underwater observation). For each survey (Supplementary material 3), we collected information on the location (island, site: approximate latitude and longitude), date of sighting (i.e., the season: winter, spring, summer, and autumn), depth, number of fish and estimated total length (TL), and type of habitat (categorized as: rocky bottoms, sandy substrates, seagrass meadows, or mixed sandy‐rocky bottoms). The size of sharks was then categorized as juvenile and subadults (<70 cm TL) and adults >70 cm, according to the sexual maturity size of the species (Muus & Nielsen, 1999). Interviewers covered all islands to have a balanced effort across the archipelago (ca. 20 questionaries per island). It is worth noting that, despite the western islands being less populated than the central and eastern islands, SCUBA diving is of great popularity at the westernmost island (El Hierro), with nine diving centers and > 20,000 divers per year, which counteracts the possible low observation effort at the western islands (Meyers et al., 2017). A total of 14 surveys did not account for any observation. The effort, as the total number of hours of observation for each year, from 1980 to 2020, at each island, was then estimated. All sightings were then standardized according to the observation effort (number of hours per year) to provide a SPUE (sightings per unit effort) for each island.

### 2.3 Shark presence through LEK: Gastronomic heritage

This fish product is locally consumed both fresh and dried‐salted, following several recipes (González, 2016; González, 2020). When dried and salted, fillets are cut in strips, locally known as “tollos,” the most common product. At each island, a survey (Supplementary material 4) was distributed among stakeholders (*N* = 28, 4 surveys per island) involved in local fisheries and commercialization of fish products (fishers and deckhands of artisanal boats, restaurant owners, and chefs). In brief, we compiled information on gastronomic ways (number of recipes) to cook *M. mustelus*, either as fresh or dried‐salted at each of the seven major Canarian islands.

### 2.4 Shark presence through fisheries landings

Despite *M. mustelus* not being one of the main target species of artisanal fisheries, this species accounts for 52.94% of the total elasmobranch captures of the artisanal trammel net fishery in the archipelago (Mendoza et al., 2018). This shark is very scarce in captures by recreational fishers, particularly spearfishers (Jiménez‐Alvarado et al., 2020). We compiled data on annual landings of *M. mustelus* at each island of the archipelago, through the 2007–2019 period, via the regional fisheries authority (www.gobiernodecanarias.org/pesca/). Most artisanal fishers operate with a range of fishing gears, mainly traps, hooks‐and‐lines and trammel nets at insular scales, using small‐sized boats (~9 m in length and 40 HP, González, González‐Lorenzo, et al., 2020). Data were then standardized according to the number of artisanal boats per island (Table 1), to control for varying fishing effort among islands; this has been previously implemented in the archipelago to assess the effect on coastal fishery resources, such as parrotfishes and groupers (Tuya, Sánchez‐Jerez, & Haroun, 2006).

### 2.5 Statistical analyses

All statistical modeling and testing were implemented in the R_4.0.2_ statistical environment (R Core Team). A t‐test checked whether the mean depth at which adults were sighted differed from the mean depth at which juveniles and subadults were sighted. Contingency tables and associated χ^2^ tests checked for differences in the proportions of sightings according to the seasons and habitats of sightings, separately for juveniles and subadults and adults, respectively, for the overall study. Mixed‐effects Generalized Linear Models (GLMs) were fitted to the number of sightings and annual fisheries landings, by means of the “lmerTest” R package (Kuznetsova et al., 2017), to test for differences among the three island groups (eastern, central, and western islands), as a fixed factor, and years and islands within each group as random factors. A mixed‐effects GLM also tested for differences in the number of recipes among island groups, as a fixed factor, and islands within each group, as a random factor. All models were fitted using a “negative binomial” family distribution of residuals, with a “log” link function, which is robust for overdispersed data. Diagnosis plots of residuals and Q–Q plots were visually inspected to check the appropriateness of the fitted models (Harrison et al., 2018). We used the function “relevel” to run models with varying reference levels to assess significant differences between each pair of island groups.

## 3 RESULTS

### 3.1 Shark presence and population structure through LEK: Sightings

**FIGURE 2:**
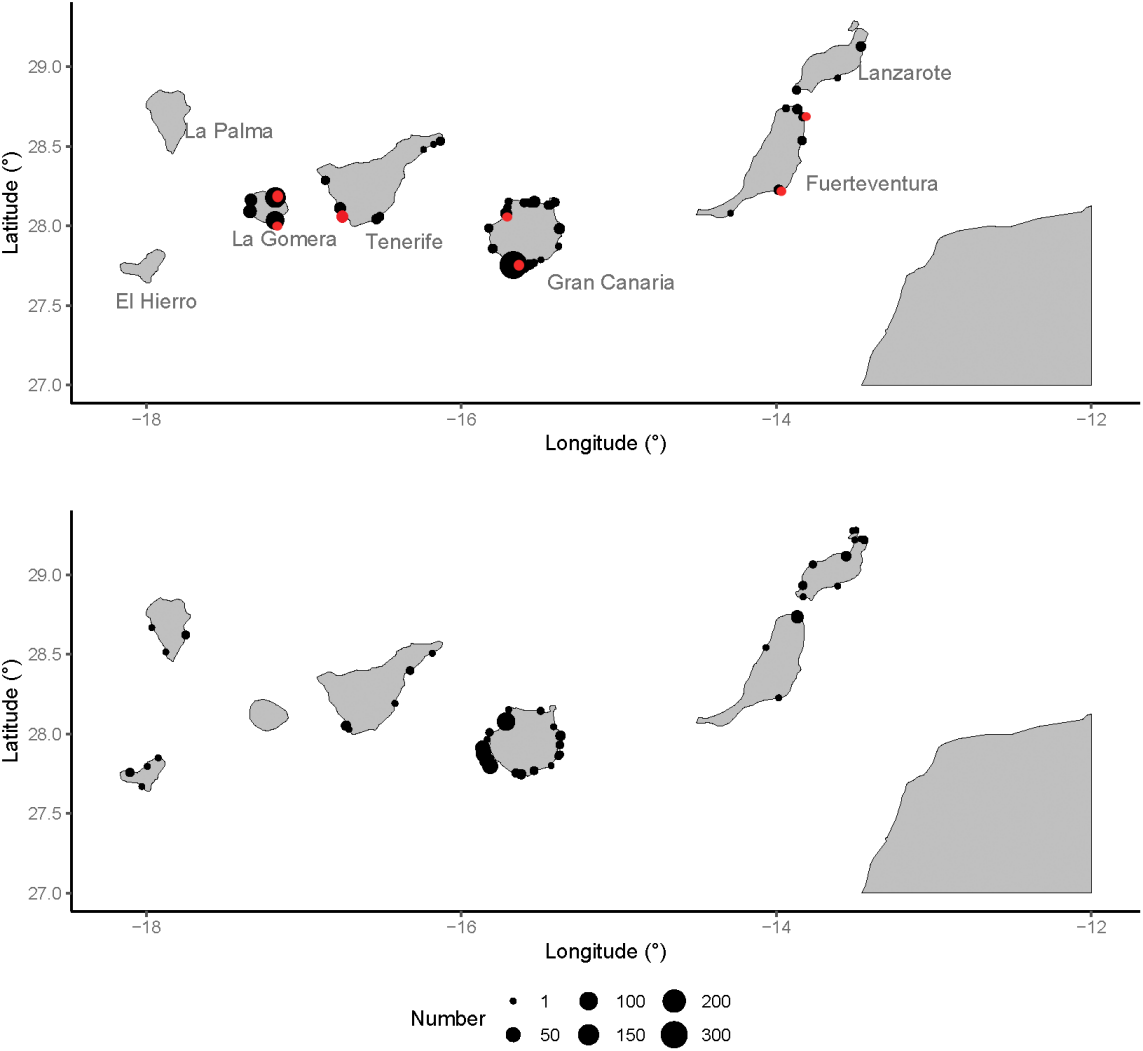
Number of sightings for juveniles and subadults (top) and adults (bottom) across the entire Canary Islands. The red dots denote spots of juvenile aggregations, where groups of juvenile sharks have been observed at least 2 years (except for the island of La Gomera, see results of sighting)

The presence of juveniles and subadults was lower in the western than in both the central and eastern islands (Figure 2; Table 2), despite the fact that results were not statistically significant because of the large random variation (Table 3). Similarly, the abundance of adults was significantly larger in the eastern and central islands of the archipelago than in the western islands (Figure 2, Table 3).

**TABLE 2:** Mixed‐effects GLM testing for the effect of “Island groups” on sightings of juvenile and subadults *M. mustelus*. the model contains two random effects (“year” and “island”) for a model of “only‐random” intercepts

Random effects	Variance	SD
Year (intercept)	0.534	0.7308
Island (intercept)	4.918	2.2176

Fixed effects (reference level = Central I.)	Estimate	SE	Z value	*p*‐value
Intercept	1.8710	1.5993	1.170	.242
Eastern I.	−0.5303	2.2697	−0.234	.815
Western I.	−3.0355	2.3735	−1.279	.201
(reference level = Eastern I.)				
Intercept	1.3407	1.6359	0.82	.412
Central I.	0.5303	2.2701	0.234	.815
Western I.	−2.5053	2.4094	−1.040	.298

**TABLE 3:** Mixed‐effects GLM testing the effect of “Island groups” on sightings of adults *M. mustelus*. The model contains two random effects (“Year” and “Island”) for a model of “only‐random” intercepts. Significant *p*‐values are highlighted in bold

Random effects	Variance	SD
Year (intercept)	9.951e^−10^	3.154e^−05^
Island (intercept)	3.538e^−01^	5.948e^−01^

Fixed effects (reference level = Central I.)	Estimate	SE	Z value	*p*‐value
Intercept	1.9921	0.5340	3.731	**.000191**
Eastern I.	−0.4873	0.7190	−0.678	.497923
Western I.	−1.9395	0.7857	−2.469	**.013564**
(reference level = Eastern I.)				
Intercept	1.9584	0.6141	3.189	**.00143**
Central I.	−0.5054	0.7551	−0.669	.50329
Western I.	−2.0609	0.9074	−2.271	**.02314**

**FIGURE 3:**
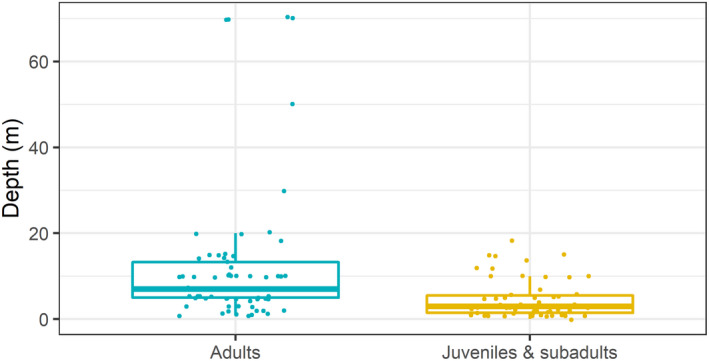
Depth (m) at which juveniles and subadults and adults were sighted according to questionaries. Each point corresponds to the mean depth identified by an interview

A total of 1254 juveniles and subadults were reported from the surveys. The size (TL) of juveniles and subadults varied between 30 and 65 cm, with a mean size (± SE) of 40 ± 0.07 cm. The mean depth of these observations was 4.66 ± 4.46 m (Figure 3). A total of 549 adults were reported, which ranged in size (TL) between 70 and 190 cm, with a mean size of 97 ± 0.08 cm. The mean depth at which adults were sighted was 12.77 ± 16.91 m, which was significantly larger than the mean depth at which juveniles and subadults were sighted (Figure 3; t‐test = 3.65, df = 72.531, *p* = .00047).

**FIGURE 4:**
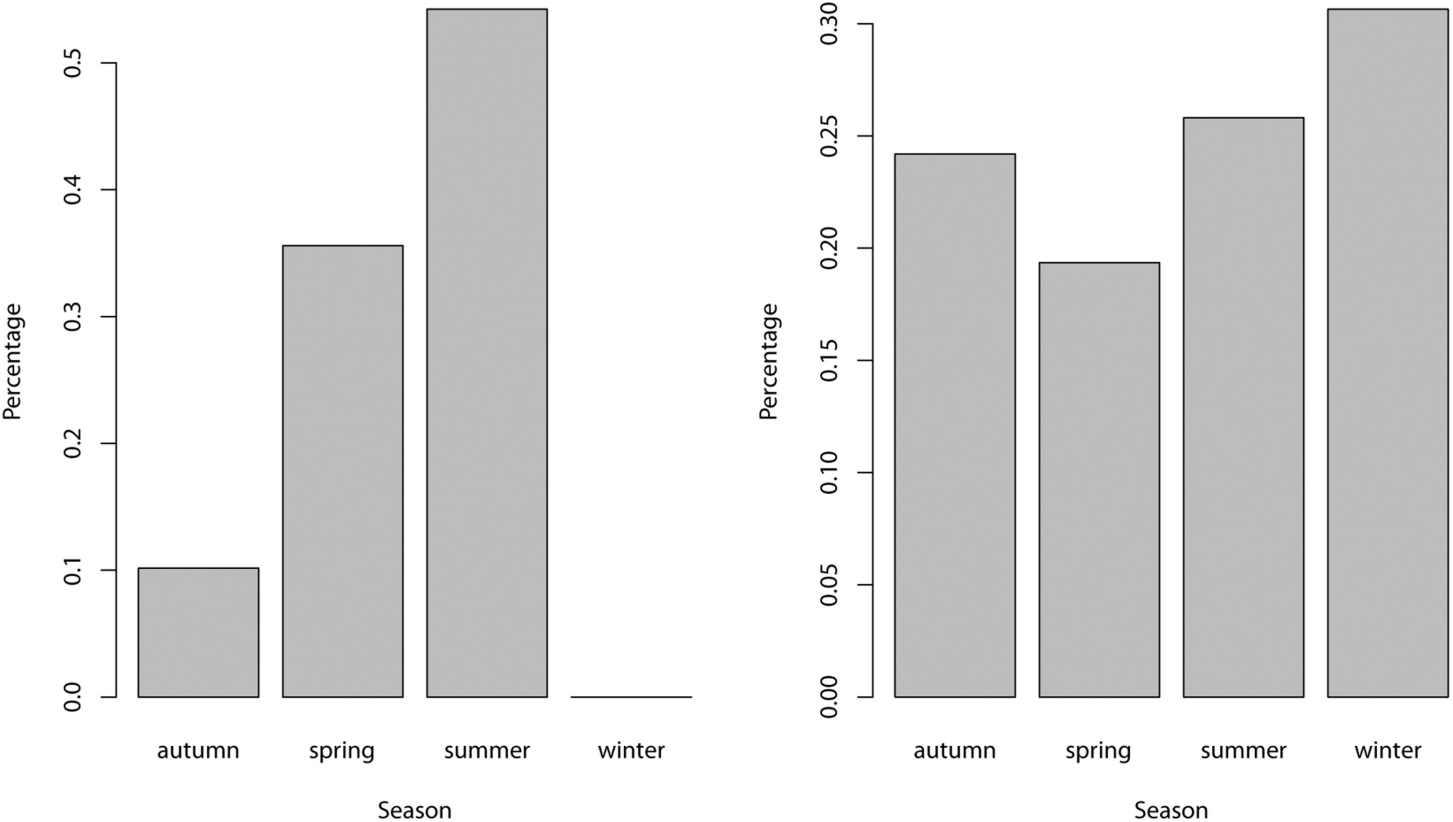
Number of sightings per season for juveniles and subadults (left) and adults (right). Data pooled for the entire Canary Islands

**FIGURE 5:**
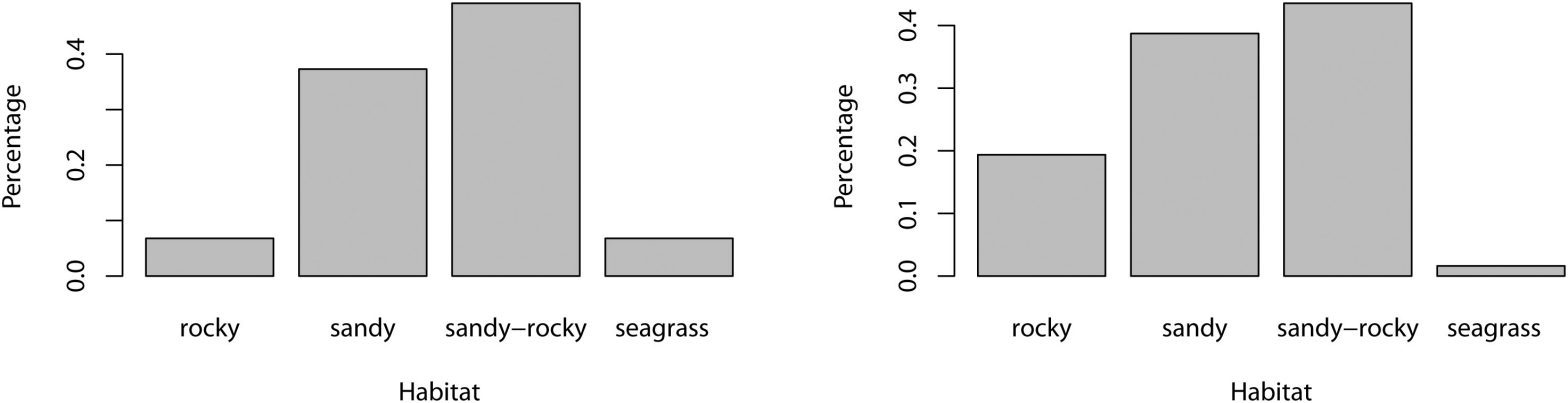
Number of sightings per habitat for juveniles and subadults (left) and adults (right). Data pooled for the entire Canary Islands

Juveniles and subadults were predominantly observed in spring (April, May, and June) and summer (July, August, and September) (ca. 89%, Figure 4, χ^2^ = 25.983, *p*‐value = 2.28e^−06^). However, adults were reported to occur throughout the entire year (Figure 4, χ^2^ = 2.15, *p*‐value = .5418). Both juveniles and subadults (χ^2^ = 44, *p*‐value = 1.509e^−09^), as well as adults (χ^2^ = 33.459, df = 3, *p*‐value = 2.576e^−07^), were commonly spotted on sandy and mixed rocky‐sandy bottoms (Figure 5). Importantly, seven aggregation areas of juveniles and subadults have been identified: two in Fuerteventura Island (Playas de Corralejo, 28°44′02.12”N, 13°51′57.26”W and Las Playitas 28°13′38.78”N, 13°59'04.62”W); two in Gran Canaria (Playa de Santa Agueda, 27°45′16.50”N, 15°22′11.10”W and Playa de la Salinilla, 28°04′43.63”N, 15°42′54.72”W); two in La Gomera Island (Playa de Hermigua, 28°10′44.03”N, 17°10′48.78”W and Playa de Tapahuga, 28°02′06.42”N, 17°10′56.84”W); and one in Tenerife Island (Playa de Las Vistas, 28°02′58.13”N, 16°43′32.89”W). All of these areas have been used at least once at two different years by juveniles and subadults, except in La Gomera Island where they have only been used once.

### 3.2 Shark presence through LEK: Gastronomic heritage

**FIGURE 6:**
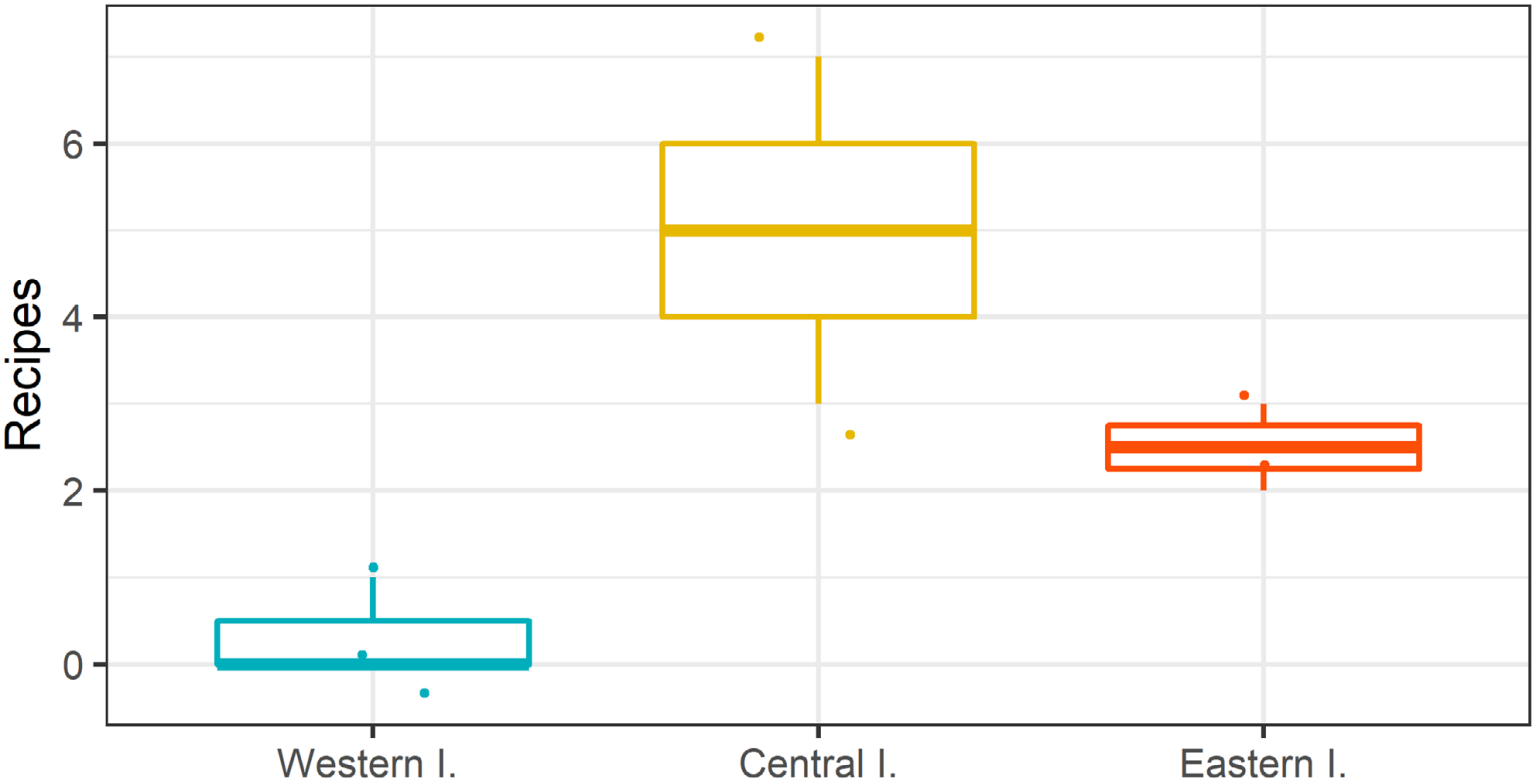
Number of recipes to cook *Mustelus mustelus* per group of islands. Each point corresponds to an island

The number of recipes (Supplementary material 5) was larger in the central and eastern islands than in the western islands (Figure 6, Table 4). Despite the number of recipes being similar (four and five, respectively) for a product based on fresh flesh, or as a dried‐salted flesh, recipes based on fresh flesh were observed in only two islands, while recipes based on a processed dried‐salted product were detected in five islands (Supplementary material 5).

**TABLE 4:** Mixed‐effects GLM testing the effect of “Island groups” on the number of recipes to cook *M. mustelus*. The model contains one random effect (“Island”) for a model of “only‐random” intercepts. Significant *p*‐values are highlighted in bold

Random effects	Variance	SD
Island (intercept)	6.659e^−12^	2.581e^−06^

Fixed effects (reference level = Central I.)	Estimate	SE	Z value	*p*‐value
Intercept	1.6094	0.3160	5.094	**3.51e** ^**−07**^
Eastern I.	−0.6931	0.5464	−1.269	.2046
Western I.	−2.7080	1.0543	−2.569	**.0102**
(reference level = Eastern I.)				
Intercept	0.9163	0.4446	2.061	**.0393**
Central I.	0.6932	0.5453	1.271	.2037
Western I.	−2.0149	1.0748	−1.875	.0608

### 3.3 Shark presence through fisheries landings

**FIGURE 7:**
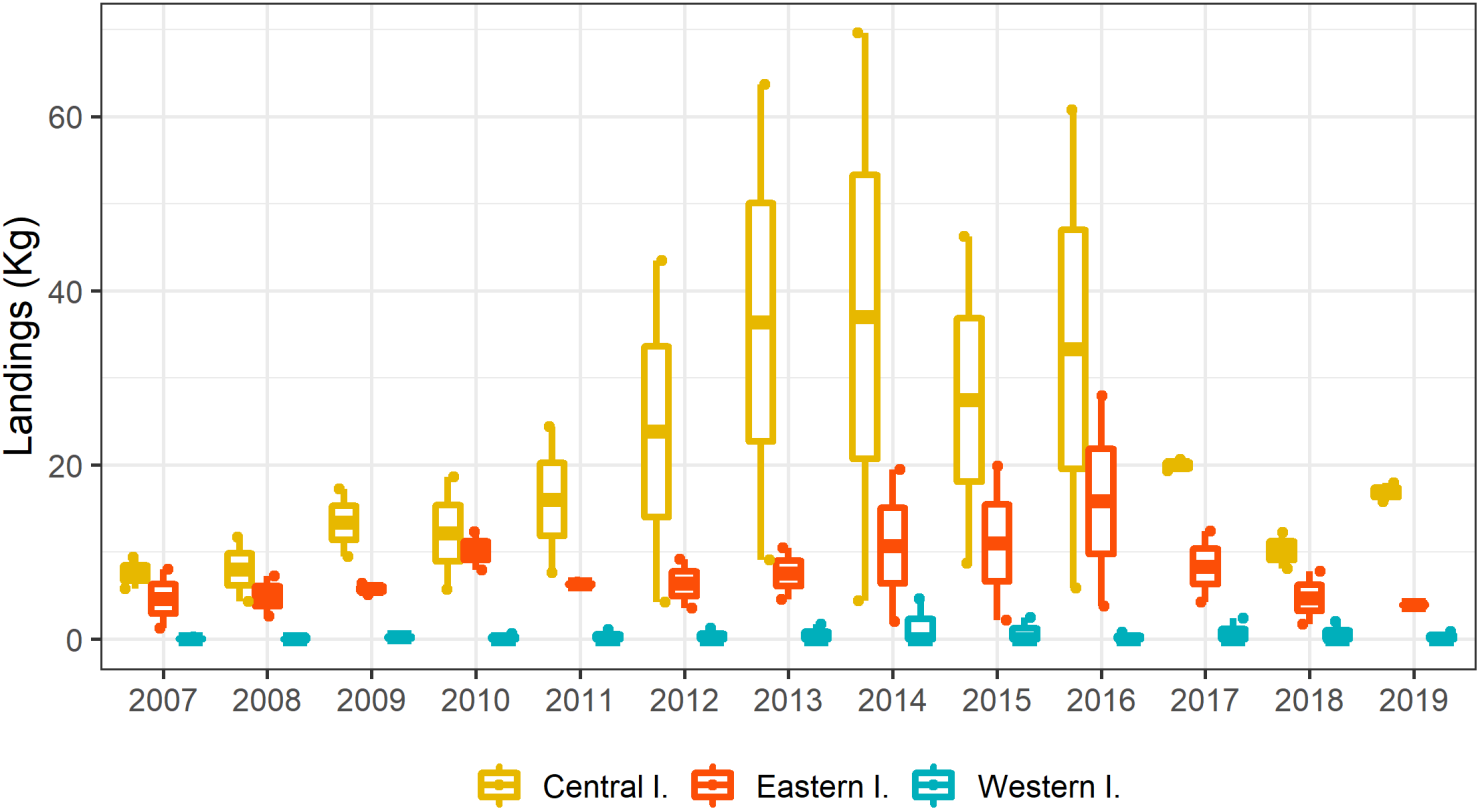
Annual landings (kg per artisanal boat through 2007 to 2019) of *Mustelus mustelus* at each of the three group of islands of the Canarian archipelago. Each point corresponds to an island and year

At the westernmost islands, landings of *M. mustelus* were close to zero (Figure 7). Consistent, but annually variable (i.e., between years), landings were otherwise observed at the easternmost and central islands of the archipelago (Figure 7). This resulted in statistically significant differences in landings between the eastern and the central islands, relative to the western islands (Table 5).

**TABLE 5:** Mixed‐effects GLM testing the effect of “Island groups” on fishery landings of *M. mustelus*. The model contains two random effects (“Year” and “Island”) for a model of “only‐random” intercepts. Significant *p*‐values are highlighted in bold

Random effects	Variance	SD
Year (intercept)	0.0283	0.1682
Island (intercept)	1.2858	1.1339

Fixed effects (reference level = Central I.)	Estimate	SE	Z value	*p*‐value
Intercept	2.7831	0.8119	3.428	**.0006**
Eastern I.	−0.8134	1.1472	−0.709	.4783
Western I.	−4.6685	1.2264	−3.807	**.0001**
(reference level = Eastern I.)				
Intercept	1.9697	0.8138	2.420	**.0155**
Central I.	0.8134	1.1472	0.709	.4731
Western I.	−3.8552	1.2279	−3.140	**.0017**

## 4 DISCUSSION

This study has demonstrated that the smooth‐hound shark, *Mustelus mustelus*, presents a gradient‐type distribution across the Canarian archipelago, with a larger presence in the eastern and central than in the western islands. Such a distribution pattern has been consistent for the two sources of biological data: LEK, through surveys on sightings and recipes, as well as from fisheries landings. These results highlight the importance of adequate regional management and conservation plans for this species at different geographic scales (Maduna et al., 2016). The heterogenous distribution pattern may be related to the dispersal limitation of the species.

In the Canary Islands, the older islands are located in the eastern and central part of the archipelago (Table 1), so these islands have wider insular shelfs relative to the most recent islands (La Palma and El Hierro, in particular, see Table 1), as a result of the long‐time presence of erosion agents (Mitchell et al., 2003). In this sense, there is a priori more suitable habitat for smooth‐hound sharks in the eastern and central compared with the western islands. Our data indicated that *M. mustelus* preferentially uses sandy and mixed (sandy‐rocky) bottoms, similar to previous observations from this (Brito et al., 2002). The same has been observed in other regions along the species' distributional range, such as South Africa and Senegal (Capapé et al., 2006; Smale & Compagno, 1997). Extensive soft bottoms on insular shelfs of the eastern and central islands, therefore, provide an explanation for the larger presence of this shark in these islands. At the same time, the eastern and central islands are closer to the adjacent African coasts. Proximity to the African coast has been pointed out to influence past and present colonization events by other benthic sharks across the archipelago, for example, to explain the decrease of angelshark, *Squatina squatina*, occurrences towards the westernmost islands (Meyers et al., 2017). The proximity of the Western African Upwelling area also promotes an east to west gradient in planktonic life and associated fish fauna (Valdés & Déniz‐González, 2015).

The dispersion potential of a species depends on its life history traits, such as the reproduction type. *Mustelus mustelus* is a viviparous (live‐bearing) species with direct development of embryos inside the mother; female sharks release their offspring in very shallow waters along coastal areas (De Maddalena et al., 2001). This reproduction mode notoriously limits the dispersion capacity of this shark (Bone & Moore, 2008; da Silva, 2018). In turn, recent genetic studies have shown strong genetic variation of *M. mustelus* along its geographical distribution range. This is the case for populations from the Mediterranean Sea, and the west and southern African coasts, including fine‐scale population structure in each region, but a lack of correlation between genetic and geographical distance (Hull et al., 2019). A similar outcome of genetic differentiation (large genetic variation) was observed between populations from the south‐western Indian and south‐eastern Atlantic Oceans (Bitalo et al., 2015; Maduna et al., 2016). As a result, the dispersion of this species is likely to be the result of adult movements. Despite being an epibenthic and demersal species, majorly living on continental shelfs, adults have been also observed, on a few occasions, swimming in the water column (Compagno, 1984). This capacity may explain the colonization of oceanic archipelagos in the eastern Atlantic not far away from the African continent, for example, the Canary Islands, São Tomé and Príncipe, and Cape Verde Islands, where *M. mustelus* is one of the most frequently captured shark species (González, Monteiro, et al., 2020; Lopes et al., 2016; Mendoza et al., 2018). In Madeira archipelago, the smooth‐hound shark is seen around the islands all year round (Biscoito et al., 2018; Martínez‐Escauriaza et al., 2020). It is worth noting, however, that this species is absent in those Atlantic oceanic archipelagos far away (> 800 km) from the nearby continental masses, for example, Azores Islands, Ascension and St. Helena Islands (Barcelos et al., 2021; Brown et al., 2019; Wirtz et al., 2017). In brief, these observations point towards a limited capacity of *M. mustelus* to overcome abyssal barriers, likely enhanced by a sequential colonization of nearby islands from the continental masses, that is, stepping‐stones that favors the colonization of islands across oceanic archipelagos (Mazzei et al., 2021).

In this study, we have used varying data sources. Overall, we are confident about the outcomes of questionaries and fisheries landings, mostly because both sources of data pointed in the same direction, that is, there were more sharks in the central and eastern, relative to the western islands. Survey respondents encompassed a range of backgrounds, from fishers to divers and marine scientists. The varying range of backgrounds was important to avoid biased surveys from particular groups. It is obvious that underwater visual estimates by divers may have some degree of uncertainty, particularly fish size. Still, these shortcomings are minored to some extend because all surveyors were highly experienced (>20 years). Fisheries landings come from official fisheries statistical data, which were temporally consistent (unpublished data) among islands, so reinforcing evidence for spatial patterns. According to the fisheries legislative frameworks, at both the national (Law 3/2001) and regional levels (Law 17/2003), all maritime professional fishing activities should land their catches in authorized harbors through official fish markets. All captures are weighted and labeled there, including sharks. All data we have used in this work were provided by the official fishing authority of the Canary Islands, so we are confident about most captures of *M. mustelus* have been reported. Still, some illegal poaching is always possible.

In addition to short‐term views and observations of a living generation, LEK may provide a cumulative body of knowledge transferred through generations by cultural transmission, which reflect the relationship of fauna with their environment (Hiddink et al., 2019). In our case study, we have shown that an ecological pattern has an imprint on local gastronomic legacies, with a larger number of recipes in the eastern and central, relative to the western islands. In this sense, the gastronomic heritage is a way of transmitting the cultural value of the fishery resource, contributing in some way to the recognition of the quality of the resource, to the need for its sustainable use and ultimately to the conservation of the species. The use of local raw materials (e.g., fish) that identifies with the regional gastronomic identity has been also observed for other coastal areas (da Silva et al., 2015). As a result, inadequate management of coastal resources that lead to local extirpations may cause potential cultural (gastronomic) losses, particularly for regions whose economy is majorly tourism‐dependent, as the Canary Islands, where visitors tend to consume local (fresh) seafood products.

The data presented here suggest that the reproductive seasonality of the species is like those observed elsewhere (Capapé et al., 2006; Ould Mohamed Fall, 2002; Smale & Compagno, 1997). Overall, adults tend to congregate on shallow waters at the end of summer, most likely to mate. Smale and Compagno (1997) found that *M. mustelus*, in South Africa, do not appear to aggregate most of the time. However, here sporadic large catches of similar‐sized individuals by fishers, at the same spot in a short period of time, suggests some schooling, or at least a certain degree of aggregation, for some time; da Silva et al. (2013), also in South Africa and by means of passive telemetry, demonstrated that adult sharks concentrated on shallow waters during summer, whereas isolated sharks were widely distributed throughout the study area in winter. The gestation period of this species last approximately 1 year, varying between 9 and 15 months (Capapé et al., 2006; Saïdi et al., 2008). Hence, the aggregation of juveniles and subadults we report here occur in late spring (May–June) and early summer (July–August). These “nursery” sites correspond to some semi‐enclosed bays in very shallow waters, as those reported from the nearby Madeira Island for this species (Biscoito et al., 2018). In this type of habitats, the presence of potential predators is low, but feeding resources are abundant, as it has been reported for other nearshore sharks, also in the Canary Islands, such as the angelshark, *Squatina squatina* (Jiménez‐Alvarado et al., 2021) and several rays (Tuya et al., 2020). This species would stand to benefit from protection of these sites, which seems to play a key role in a critical life stage of *M. mustelus* in the Canary Islands, particularly since this species has a high degree of site fidelity, at least in other regions (da Silva et al., 2013; Klein et al., 2021) and observations of juvenile and subadults occur in successive years (da Silva, 2018). Most specifically, these nearshore “nursery” sites should be initially identified and then, if meeting the criteria to be considered nurseries (Heupel et al., 2007), protected of several common human perturbations, including infrastructures construction, sewage outlets, and excessive maritime traffic linked to certain tourist activities (e.g., jet skis). In addition to this, and regarding conservation implications, our results suggest that, because of the large differences in abundance of this shark among the Canary Islands, management of the species should be adapted to the specific peculiarities of each island, rather than adopting a management policy at the entire archipelago‐scale. This strategy reinforces the idea of taxon specificities, that is, taxon dependencies, when depicting conservation actions on coastal elasmobranchs in the Canary Islands (Tuya et al., 2021). This should be underpinned by more research into the species' habitat, ecology, distribution, and behavior. This should be better understood and taken into consideration to complement conservation and management strategies at each island. This may include spatial closures, or even lowering minimum size limits at certain islands relative to the regional limit, which includes a legal first size capture limit of 96 cm (total length, González et al., 2012). We also would recommend a temporal ban between April and October, when individuals concentrate in nearshore waters. These measures would help to assure sustainability of this traditional culinary resource. In brief, this study has demonstrated differences in the distribution of *M. mustelus* across an oceanic archipelago. Aggregations of juveniles in spring (April, May, and June) and summer (July, August, and September), that is, pupping, deserves confirmation through further investigation. A long‐term study that addresses basic biological parameters of the Canarian populations of this shark species, including abundance estimates and detection of nearshore aggregation sites is necessary to confirm results we here provide by means of LEK and fisheries landings.

## AUTHOR CONTRIBUTIONS

**Fernando Espino:** Conceptualization (lead); data curation (equal); writing – original draft (lead). **José Antonio González:** Data curation (equal); investigation (equal); writing – review and editing (equal). **Nestor Bosch:** Writing – original draft (equal). **Francisco Otero‐Ferrer:** Data curation (equal); writing – review and editing (equal). **Ricardo Haroun:** Writing – review and editing (equal). **Fernando Tuya:** Conceptualization (lead); data curation (equal); formal analysis (lead); writing – original draft (lead).

## CONFLICT OF INTEREST

The authors declare that they have no conflicts of interest associated with this work.

## Supporting information


**Supplementary material 1** Questionaries filled by respondents.

**Supplementary material 2** Sampling effort, in terms of number of questionaries, according to the background of survey respondents.

**Supplementary material 3** Survey to collect information on sightings.

**Supplementary material 4** Survey to collect information on recipes.

**Supplementary material 5** Registered recipes to cook *Mustelus mustelus* in the Canary Islands.

## Data Availability

Sampling locations and presence data are stored at Dryad: doi:10.5061/dryad.pvmcvdnpq.
