# Supplementary figures and images for: Distribution and population structure of the smooth‐hound shark, Mustelus mustelus (Linnaeus, 1758), across an oceanic archipelago: Combining several data sources to promote conservation
Source: Ecol Evol. 2022 Jul 13;12(7):e9098. doi: 10.1002/ece3.9098 (PMC9277611; doi:10.1002/ece3.9098)

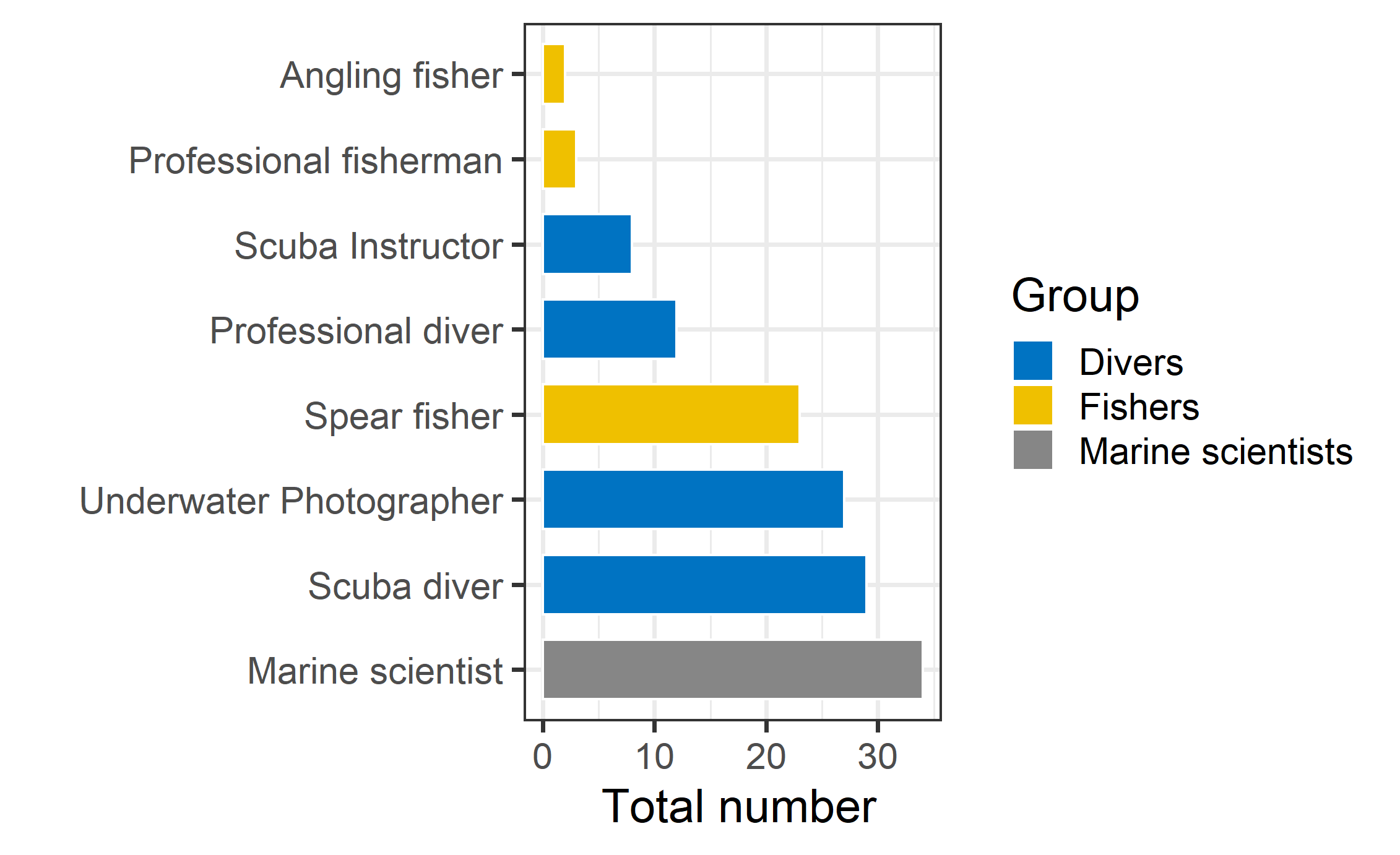

Supplement: Supplementary file 2 — Supplementary material 2 Sampling effort, in terms of number of questionaries, according to the background of survey respondents. [file ECE3-12-e9098-s005.tiff]
