# Supplementary material for: Distribution and population structure of the smooth‐hound shark, Mustelus mustelus (Linnaeus, 1758), across an oceanic archipelago: Combining several data sources to promote conservation
Source: Ecol Evol. 2022 Jul 13;12(7):e9098. doi: 10.1002/ece3.9098 (PMC9277611; doi:10.1002/ece3.9098)

**Supplementary material 3**. Survey (in Spanish) to collect information on the presence of *Mustelus mustelus* across the Canary Islands, including location (island and site; approximate latitude and longitude), date of sighting (i.e., the season: winter, spring, summer and autumn), depth, number of fish and estimated total length, and type of habitat where sharks were spotted.


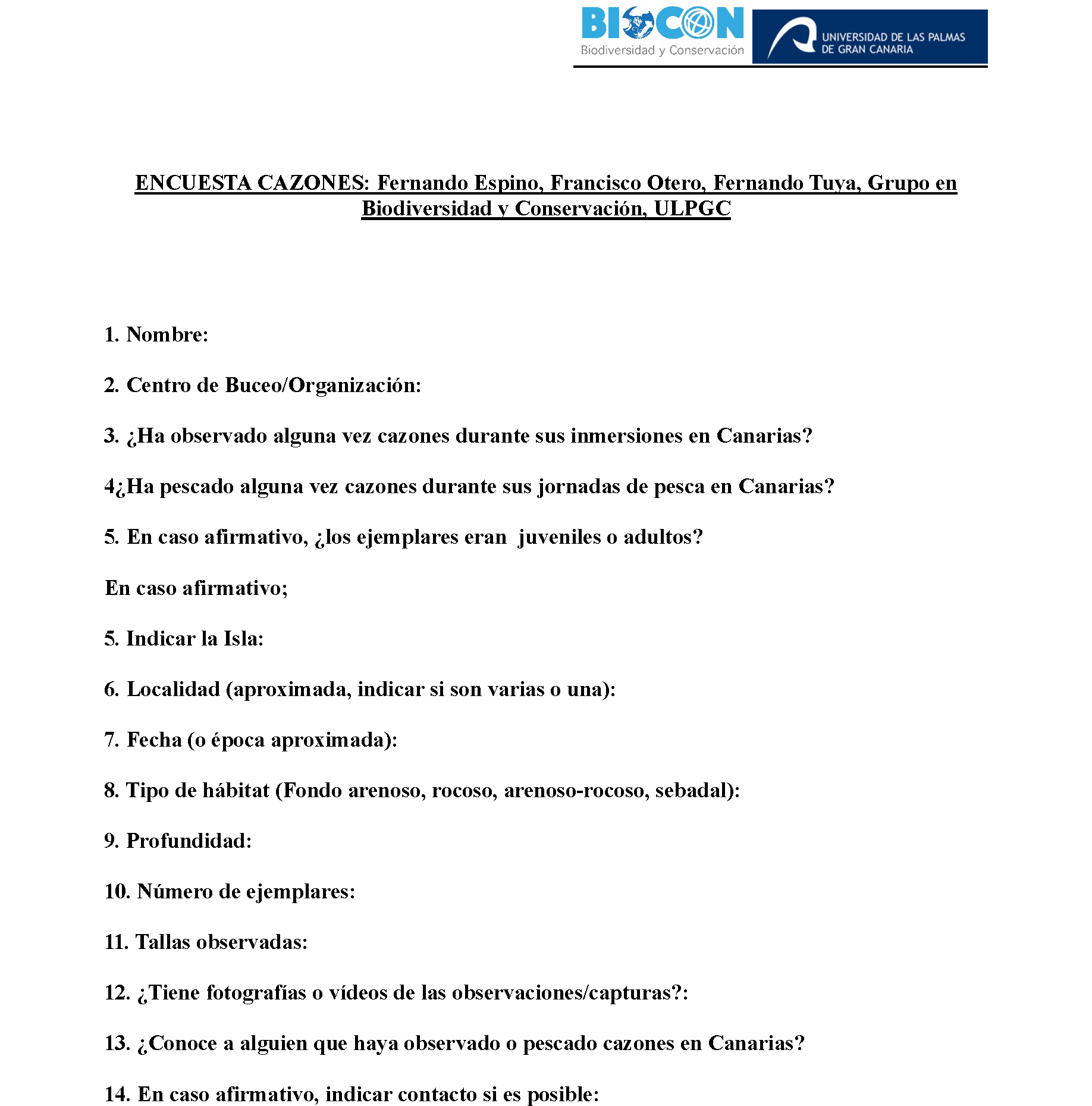

Supplement: Supplementary file 3 — Supplementary material 3 Survey to collect information on sightings. [file ECE3-12-e9098-s003.docx]
