# Supplementary material for: Distribution and population structure of the smooth‐hound shark, Mustelus mustelus (Linnaeus, 1758), across an oceanic archipelago: Combining several data sources to promote conservation
Source: Ecol Evol. 2022 Jul 13;12(7):e9098. doi: 10.1002/ece3.9098 (PMC9277611; doi:10.1002/ece3.9098)

**Supplementary material 4**: Survey (in Spanish) to collect information on recipes to cook *Mustelus mustelus* across the Canary Islands, including location (island and site), number of local boats dedicated to demersal fisheries, season of the gastronomic offer, number of recipes.


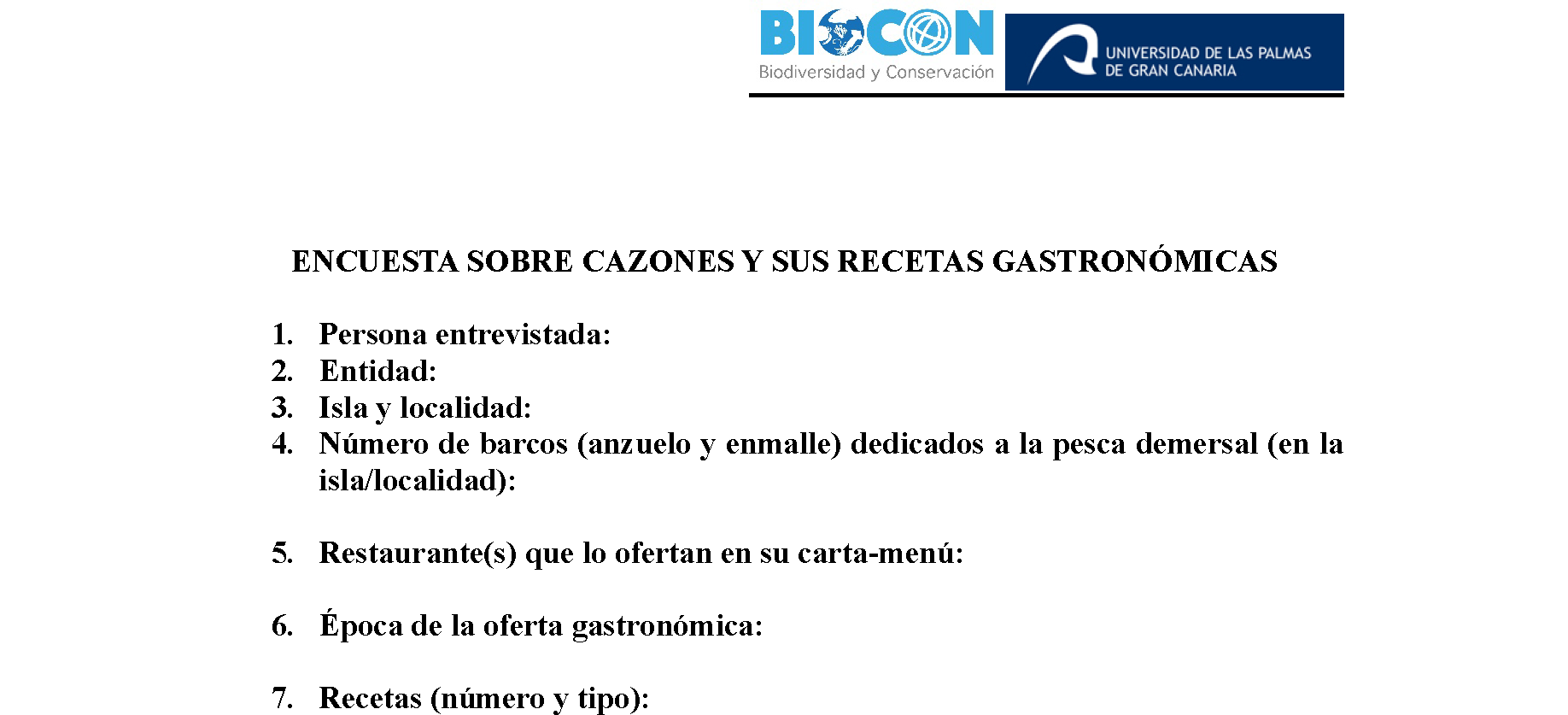

Supplement: Supplementary file 4 — Supplementary material 4 Survey to collect information on recipes. [file ECE3-12-e9098-s004.docx]
