# Supplementary material for: Distribution and population structure of the smooth‐hound shark, Mustelus mustelus (Linnaeus, 1758), across an oceanic archipelago: Combining several data sources to promote conservation
Source: Ecol Evol. 2022 Jul 13;12(7):e9098. doi: 10.1002/ece3.9098 (PMC9277611; doi:10.1002/ece3.9098)
Supplement: Supplementary file 5 — Supplementary material 5 Registered recipes to cook Mustelus mustelus in the Canary Islands. [file ECE3-12-e9098-s002.docx]

**Supplementary material 5:** Registered recipes (in Spanish, grey cells) to cook *Mustelus mustelus*, either based on fresh flesh or dried-salted, in each of the seven major islands of the Canarian archipelago.

|  |  | **Western Islands** | | | **Central Islands** | | **Eastern Islands** | |
| --- | --- | --- | --- | --- | --- | --- | --- | --- |
| **Based on fresh flesh** | | **El Hierro** | **La Palma** | **La Gomera** | **Tenerife** | **Gran Canaria** | **Fuerteventura** | **Lanzarote** |
| *Cazón en salsa con papas bonitas* | |  |  |  |  |  |  |  |
| *Bienmesabe de cazón* | |  |  |  |  |  |  |  |
| *Cazón en adobo malagueño* | |  |  |  |  |  |  |  |
| *Taquitos de cazón en adobo* | |  |  |  |  |  |  |  |
| **Based on dried-salted flesh** | |  |  |  |  |  |  |  |
| *Tollos con mojo hervido* | |  |  |  |  |  |  |  |
| *Tollos con mojo rojo palmero* | |  |  |  |  |  |  |  |
| *Tollos rebozados* | |  |  |  |  |  |  |  |
| *Tollos a la romana* | |  |  |  |  |  |  |  |
| *Tollos a la portuguesa* | |  |  |  |  |  |  |  |
| *Tollos hervidos con aceite y vinagre* | |  |  |  |  |  |  |  |
| *Ropavieja de tollo* | |  |  |  |  |  |  |  |
